# Inhibitory Effect of Bridged Nucleosides on *Thermus aquaticus* DNA Polymerase and Insight into the Binding Interactions

**DOI:** 10.1371/journal.pone.0147234

**Published:** 2016-01-28

**Authors:** Sung-Kun Kim, Aaron Castro, Edward S. Kim, Austin P. Dinkel, Xiaoyun Liu, Miguel Castro

**Affiliations:** 1 Northeastern State University, Department of Natural Sciences, Tahlequah, Oklahoma, United States of America; 2 Bio-Synthesis Inc, Lewisville, Texas, United States of America; Institute of Molecular Genetics IMG-CNR, ITALY

## Abstract

Modified nucleosides have the potential to inhibit DNA polymerases for the treatment of viral infections and cancer. With the hope of developing potent drug candidates by the modification of the 2’,4’-position of the ribose with the inclusion of a bridge, efforts were focused on the inhibition of *Taq* DNA polymerase using quantitative real time PCR, and the results revealed the significant inhibitory effects of 2’,4’-bridged thymidine nucleoside on the polymerase. Study on the mode of inhibition revealed the competitive mechanism with which the 2’,4’-bridged thymidine operates. With a *K*_i_ value of 9.7 ± 1.1 μM, the 2’,4’-bridged thymidine proved to be a very promising inhibitor. Additionally, docking analysis showed that all the nucleosides including 2’,4’-bridged thymidine were able to dock in the active site, indicating that the substrate analogs reflect a structural complementarity to the enzyme active site. The analysis also provided evidence that Asp610 was a key binding site for 2’,4’-bridged thymidine. Molecular dynamics (MD) simulations were performed to further understand the conformational variations of the binding. The root-mean-square deviation (RMSD) values for the peptide backbone of the enzyme and the nitrogenous base of the inhibitor stabilized within 0.8 and 0.2 ns, respectively. Furthermore, the MD analysis indicates substantial conformational change in the ligand (inhibitor) as the nitrogenous base rotated anticlockwise with respect to the sugar moiety, complemented by the formation of several new hydrogen bonds where Arg587 served as a pivot axis for binding formation. In conclusion, the active site inhibition of *Taq* DNA polymerase by 2’,4’-bridged thymidine suggests the potential of bridged nucleosides as drug candidates.

## Introduction

DNA replication is an essential process for the proliferation of all forms of life. The importance of the inhibition of DNA replication is hardly understated in modern therapeutic strategy against numerous diseases including viral infections and cancer. One potential venue to inhibit DNA replication is in the active site of DNA polymerase, which can be classified into at least five different families based on amino acid sequence comparison and crystal structure analyses [1,2]. Regardless of the different families, all structures of the different DNA polymerases appear to share a common overall architectural feature; they all share a general shape characterized by a thumb domain, a palm domain, and several finger domains, where the thumb and the finger domains lead to distinctions [3,4]. The palm domain serves as a catalysis site of the phosphoryl transfer reaction, the finger domains serve as the interaction site with the incoming nucleoside triphosphate and the template bases to which it will be paired, and the thumb domain serves as the positioning site of the duplex DNA and plays a key role in the process of translocation [4].

Several agents have been developed to inhibit DNA replication, for instance, chemotherapeutic agents that modify the structure and composition of DNA such as Temozolomide and Cisplatin [5] and antiviral agents that mimic nucleosides such as 3-azidothymidine (AXT) [6] and fludaribine [7]. As the antiviral drug Acyclovir, a guanosine mimic and suicide inhibitor of *Herpes simplex* virus, binds to the active site of the viral DNA polymerase [8]. However, the problems of these agents stem from development of resistance during long-term treatment and adverse side effects due to low selectivity [9,10]. It is thus imperative that development of novel inhibitors against DNA polymerases should continue.

**Fig 1:**
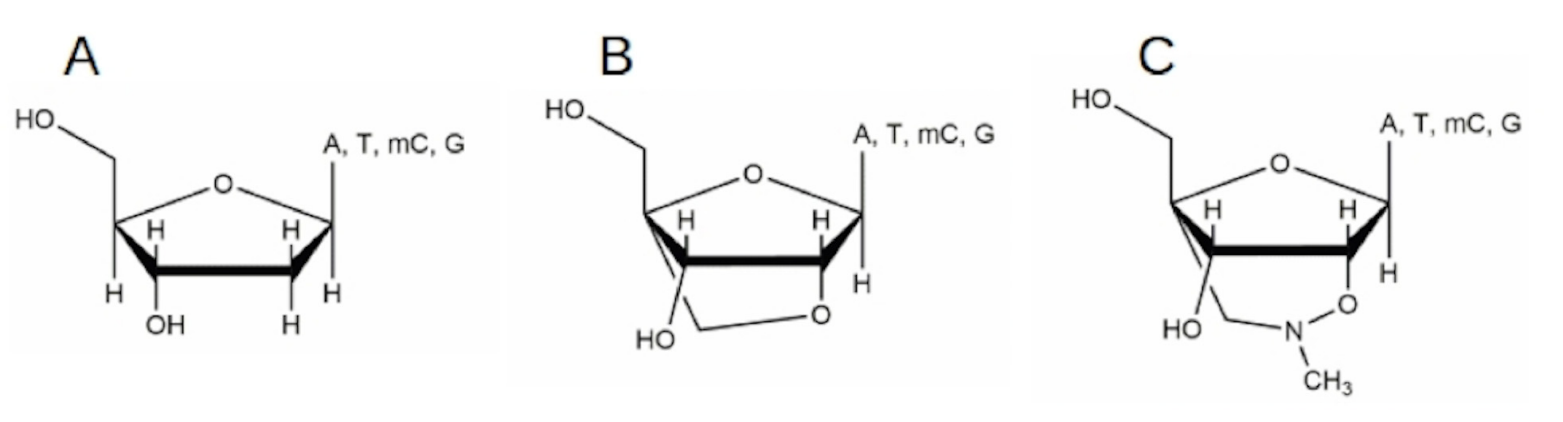
The structures of nucleosides of deoxynucleosides. (A), 2',4'-bridged nucleosides (B) and 2',4'-bridged (NC-NMe) nucleosides (C) tested for inhibition against *Taq* DNA polymerase. A represents Adenine; T, Thymine; mC, 5-Methylcytosine, G, Guanine.

In developing nucleoside or nucleotide analogs for therapeutics, modification in the sugar moiety of nucleosides has so far proven supreme [11,12,13]. After the development of dideoxy and acyclic- nucleosides or nucleotides [13], modified nucleosides called locked nucleic acids (LNAs) appeared to be very promising due to the inclusion of a bridge between 2’-O & 4’-C atom (Fig 1) [14,15]. The unique feature by the addition of the extra methylene group to the ribose moiety has been sufficiently intriguing for synthetic oligonucleotide-based therapeutic strategy; hence, LNA-containing oligonucleotides have been used for targeting the corresponding nucleic acid counterparts [14,15,16,17]. In fact, the LNA-based drug Miravirsen developed by Santaris Pharma A/S is an inhibitor of the liver specific microRNA, miR-122, which Hepatitis C virus requires for replication; the inhibitor has since entered Phase II clinical study [18]. Following the generation of LNA, bridged nucleic acids (BNAs) such as 2’,4’-BNA^COC^ and 3’-, and 5’-amino-2’,4’-BNAs were developed with enhanced binding abilities and nuclease resistance [19,20,19,21]. Since then, 2’,4’-BNA (NC-NH) and 2',4'-BNA (NC-NMe) were synthesized and have shown to be the most promising in the context of hybridization and nuclease resistant abilities [22,23,24,25].

In an attempt to explore the possibility of inhibition by the nucleoside analogs based on LNA and BNA (NC-NMe), this work used *Taq* DNA polymerase as a target polymerase, a critical component of PCR. The study demonstrated that a couple of the new nucleosides inhibit the DNA polymerase. Novel quantitative real-time PCR (qPCR) methods were developed to determine not only inhibition but also the inhibition mode. The binding interaction between the inhibitors and DNA polymerase was determined by *in silico* analysis. Furthermore, molecular dynamics (MD) simulations offered detailed binding interaction after initial docking.

## Materials and Methods

### Materials

Power SYBR Green PCR Master Mix and MicroAmp Fast 96-well Reaction Plate were purchased from Life Technologies, NY, USA. Other chemicals were purchased from Sigma (St. Louis, USA). *Taq* DNA polymerase (cat 203601) was purchased from Qiagen Inc (Valencia, USA). 2’,3’-Dideoxycytidine 5’-Triphosphate (ddCTP) was purchased from GE Healthcare Bio-Sciences (Pittsburgh, USA). Other companies providing high quality chemicals. Nucleosides were obtained from Bio-Synthesis Inc. (Lewisville, USA); that is, deoxyadenosine, thymidine, 5-methyldeoxycytidine, deoxyguanosine, 2',4'-bridged adenosine, 2',4'-bridged thymidine, 2',4'-bridged 5-methylcytidine, 2',4'-bridged guanosine, 2',4'-bridged (NC-NMe) adenosine, 2',4'-bridged (NC-NMe) thymidine, 2',4'-bridged (NC-NMe) 5-methylcytidine, 2',4'-bridged (NC-NMe) guanosine.

### qPCR using fluorescent dyes

The qPCR was performed on an Applied Biosystems StepOnePlus Real-Time PCR System (Life Technologies, NY, USA). The 10 μL PCR amplification reaction mixtures contained 5 μL of 2 x Power SYBR Green PCR Mater Mix, primers. PCR primers (200 nmol/L each) were added at a final concentration of 200 nmol/L each, where the sequences of the primers were set to be 5’- CAGGAAGCCTACGTGATG -3’ for the forward primer and 5’- CTTTGTGTTCCCGGACATAG-3’ for the reverse primer. The PCR template in which contained a part of the exon-20 sequence of the human genome was obtained from Bio-Synthesis, Inc (Lewisville, TX) and was complementary to the primers. PCR cycling was set as follows: a single cycle of DNA polymerase activation for a 10 min hold at 95°C followed by 55 cycles of 95°C for 15 s and 60°C for 1 min for annealing temp. The SYBR green channel (excitation at 470 nm, detection at 585 nm) was chosen to acquire the amplification data. The fluorescent reporter signal was normalized with the internal reference dye ROX and the threshold limit was set in automatic mode unless manual adjustment was needed. For a primer extension assay using EvaGreen, PCR amplifications were carried out in 20 μL volumes containing 10 μL 2 x EvaEZ Polymerase Activity Mix (Biotium Inc, Hayward, CA), 1 μL *Taq* DNA polymerase and various concentrations of 2’,4’-bridged thymidine or ddCTP. PCR cycling was set as 30 cycles of 72°C for 20 s to maintain the isothermal condition. All experiments were independently conducted at least twice.

### Kinetics by qPCR

In order to obtain kinetic parameters, a TaqMan method using qPCR, previously described [26], was used with modification. The sequence of TaqMan was 5’-FAM-CTGCTGGGCATCTGCCTCAC-TAMRA-3’, and the concentration of TaqMan was 125 nM for each assay reaction. The TaqMan Mix, which contains 0.2 mM dNTP, *Taq* DNA polymerase (50 U/mL), 37.5 mM MgCl_2_, 7.5 ng ROX, 25 mM Tris buffer (pH 8.0), 125 mM KCl, 5% Tween 20, was also used for each assay reaction. Due to the use of the TaqMan, the detection was set for the FAM channel (excitation at 470 nm, detection at 510 nm, extinction coefficient at 510 nm = 70,000 M^-1^cm^-1^*)*. Other conditions including the template, primers and PCR cycling remained the same as the ones described above. The increased product amounts were monitored by the qPCR, and the rate was calculated by the difference in the quantity of the amplified product over a fixed range of PCR cycles.

Data obtained by initial velocities were fit to the rate equation describing a competitive inhibition model (Eq (1)).

$$v\;=\;V_{max}[\text{S}]/([\text{S}]\;+\;K_{m}(1\;+\;[\text{I}]/K_{i}))$$ (1)

where *v* is the velocity, V_max_ represents the maximum velocity, [S] is the concentration of substrate, [I] denotes the concentration of inhibitor, K_m_ represents the Michaelis-Menten constant, K_i_ is the inhibition constant. All experiments were independently conducted at least twice.

### Molecular docking

Molecular docking between *Taq* DNA polymerase and nucleosides was performed using AutoDock Vina [27]. For the 3D *Taq* DNA polymerase structure, a 2.40 Å resolution X-ray structure (PDB code 1TAQ) was used [28]. For the 3D nucleoside structures, the MM2 force field in the ChemBio3D ver14 program was used for energy minimization. All the possible torsion angles in the nucleosides were set free to carry out flexible docking in the ligand. Polar hydrogens were added to the protein using AutoDock Tools. Gasteiger partial charges were assigned to the protein.

A grid box of 25 Å × 25 Å × 25 Å with 1.0 Å grid spacing centered on x-y-z coordinates (-3, 57.5, 81) near the potential binding pocket of *Taq* DNA polymerase was set for configuration in Autodock Vina, which uses Autodock Vina force field (based on Amber force field). The catalytic sites were assigned as flexible residues. The exhaustiveness configuration was set to 8. The results produced by the Autodock Vina showed the binding energies and hydrogen bonding interactions between the protein and nucleosides. The structure with lowest energy is chosen for computing intermolecular binding energies. In the case of T7 DNA polymerase (PDB code 1SKR), a grid box of 30 Å × 30 Å × 30 Å with 1.0 Å grid spacing centered on x-y-z coordinates (44, 22, 3) near the potential binding pocket was set for configuration in Autodock Vina; in the case of HIV-1 RT (human immunodeficiency virus-1 reverse transcriptase), a grid box of 30 Å × 30 Å × 30 Å with 1.0 Å grid spacing centered on x-y-z coordinates (48, -23, 25) near the potential binding pocket was set. All experiments were performed in triplicate.

### Molecular dynamics simulations

Molecular dynamics (MD) simulations were carried out using the GROMACS 5.0.4 package with AMBER99SB force field [29,30,31]. The topology file for the ligand was generated using ACPYPE (AnteChamber PYthon Parser interface) [32]. Ionization states were set to a neutral pH. Solvation in a cubic box was applied to the structure and the TIP3P water model was employed to produce the aqueous environment [33,34]. The systems were neutralized by adding K^+^ counterions by replacing water molecules and were added with 0.05 M KCl. The energy of the structures using the steepest descent approach was minimized with a tolerance of 1000 kJ/mol to avoid high energy interactions and steric clashes. The energy minimized system was treated for 100 ps in an equilibration run, and 1000 ps MD simulations were carried out with a time-step of 2fs at the NPT canonical ensemble and the periodic boundary conditions were used in all three dimensions. The MD simulations were run in a temperature bath at 300 K with a constant pressure of 1 bar using the V-rescale method for temperature and the Parrinello-Rahman method for pressure [35,36]. The particle mesh Ewald (PME) method for long-range electrostatics [37,38] and periodic boundary conditions were applied in all directions. A 14 Å cutoff for van der Waals interactions, a 14 Å cutoff for Coulomb interaction with updates every 10 steps, and the LINCS algorithm for covalent bond constrains were used [39]. Root mean square deviation (RMSD) values were calculated by the program GROMACS 5.0.4 and subsequently analyzed.

## Results and Discussion

**Fig 2:**
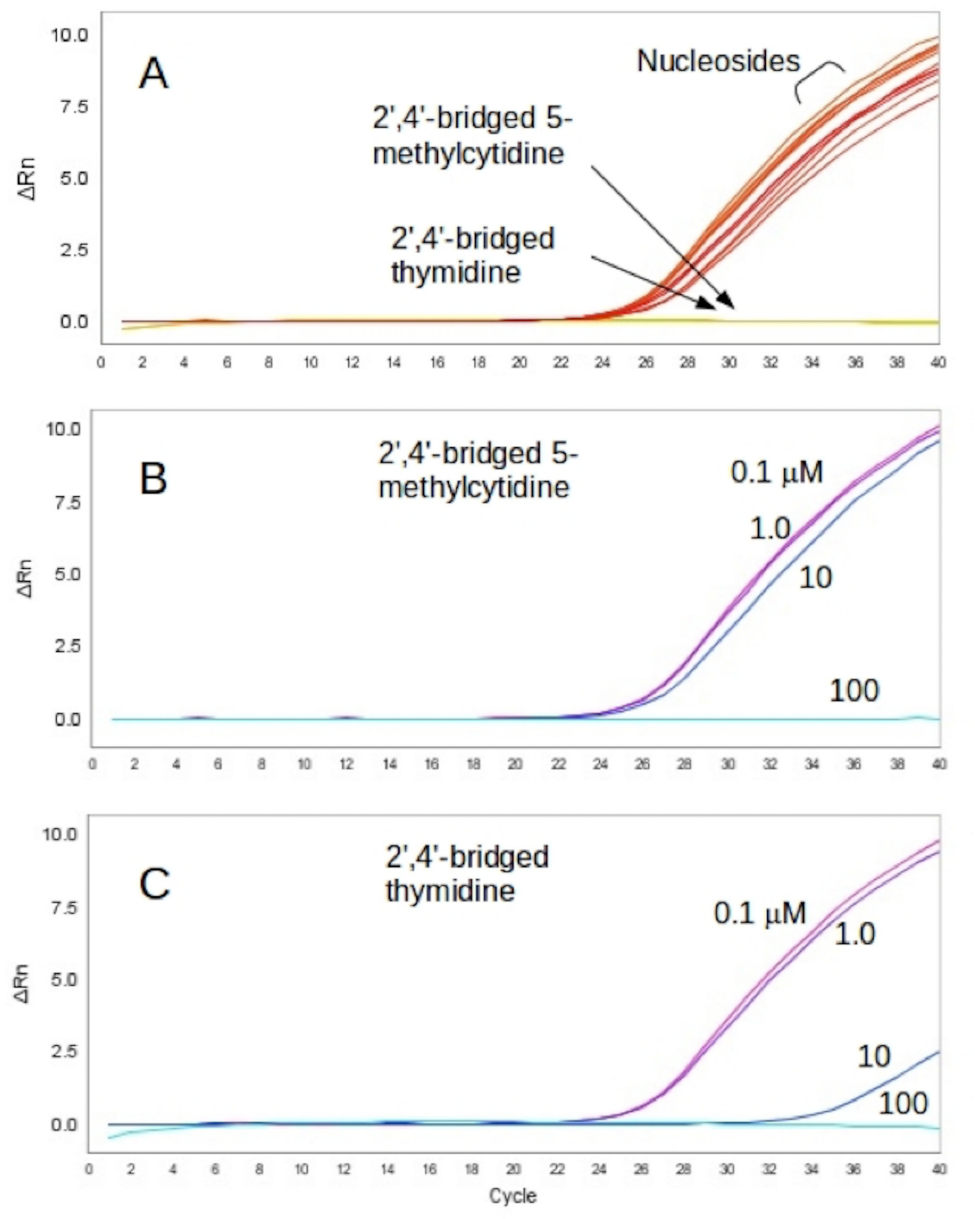
PCR product detection using SYBR Green in real time. (A) A console outcome of qPCR using nucleosides including 2',4'-bridged 5-methylcytidine and 2',4'-bridged thymidine with a concentration of 100 μM. (B) A console outcome of qPCR with various concentrations of 2',4'-bridged 5-methylcytidine. (C) A console outcome from qPCR with various concentrations of 2',4'-bridged thymidine.

An initial attempt to test inhibition for the nucleosides was made by monitoring an increase in the product amounts detected by using SYBR Green in the qPCR process. The results of 40 PCR cycles with a mixture of *Taq* DNA polymerase and 100 μM of nucleosides showed that only two nucleosides, 2',4'-bridged 5-methylcytidine and 2',4'-bridged thymidine, suppressed the amplification of PCR products as shown in Fig 2A. To further investigate how effectively the two nucleosides could inhibit *Taq* DNA polymerase, the concentrations of the two nucleosides were varied from 100 μM to 0.1 μM. In the case of 2',4'-bridged 5-methylcytidine, the 100 μM sample exhibited, as was seen in the initial testing, effective inhibition (no amplification was detected), but the 10 μM sample elicited a marginal inhibition (Fig 2B). In the case of 2',4'-bridged thymidine, a 10 μM concentration was sufficient to suppress amplification effectively as shown in Fig 2C.

**Fig 3:**
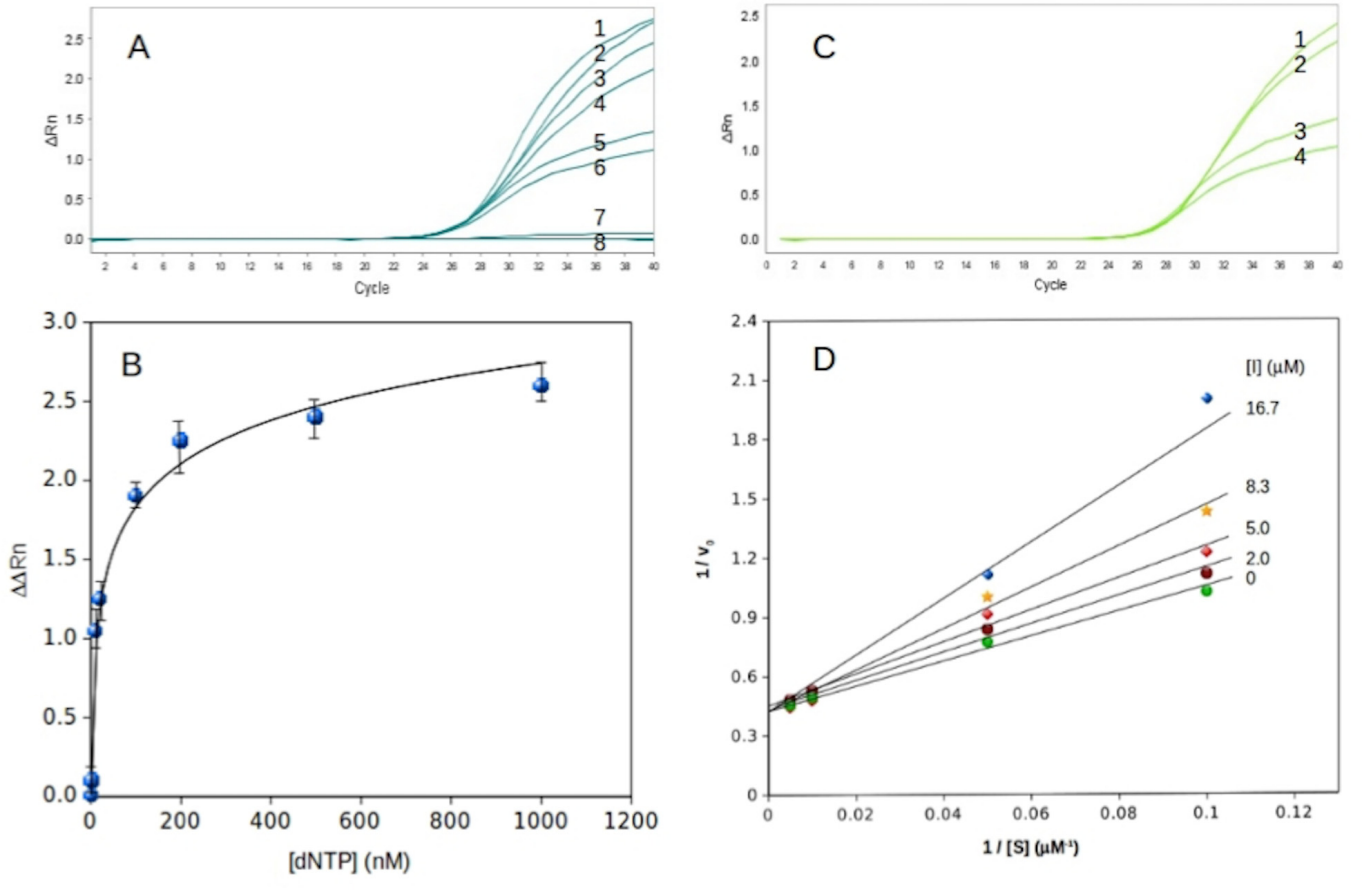
Kinetics studies of *Taq* DNA polymerase. (A) A console outcome of qPCR using various concentrations of dNTP: 1, 1000 μM; 2, 500 μM; 3, 200 μM; 4, 100 μM; 5, 20 μM; 6, 10 μM; 7, 2 μM; 8, 0 μM. (B) A Michaelis-Menten plot of the dNTP concentration. (C) A console outcome of qPCR using various concentrations of dNTP: 1, 200 μM; 2, 100 μM; 3, 20 μM; 4, 10 μM. The concentration of 2',4'-bridged thymidine used is 5 μM. (D) A double-reciprocal plot of the substrate-reaction rate relationship with various concentrations of the inhibitor 2',4'-bridged thymidine. The concentrations of dNTP used were 200 μM, 100 μM, 20 μM and 10 μM, and the concentrations of 2',4'-bridged thymidine were 16.7 μM, 8.3 μM, 5.0 μM, 2.0 μM and 0 μM.

In an attempt to further explore the inhibition by 2',4'-bridged thymidine, the initial experimentation was followed by the subsequent study of kinetic parameters of *Taq* DNA polymerase using the TaqMan method. It should be mentioned here that although SYBR Green was initially more convenient to use in testing for potential inhibition, qPCR with TaqMan, due to ingredient manipulability, was used for the later kinetic analysis to provide more detailed information. Under the TaqMan method (see the Materials and Methods section 2.3), the substrate dNTP concentration was varied at a fixed enzyme concentration at 0.5 unit/μL (50 U/mL) for each qPCR process, and the results are shown in Fig 3A. The data obtained from the qPCR were plotted as a function of the substrate concentration as shown in Fig 3B. The data fits well with respect to the Michaelis-Menten equation with *K*_m_ = 33.3 ± 2.5 μM and *V*_max_ = 2.2 ± 0.5 μM·cycle^-1^. Previous attempts by Higuchi et al. at kinetics analysis using a PCR involved the monitoring of fluorescence increase with a camera; however, this method could not offer stable data acquisition and therefore could not provide quantitative kinetic parameters [40]. With the onset of qPCR and TaqMan, more precise data acquisition opened up new possibilities for enzyme kinetics analysis. To the best of our knowledge, this is one of the first attempts at obtaining kinetic parameters using the qPCR assay.

**Fig 4:**
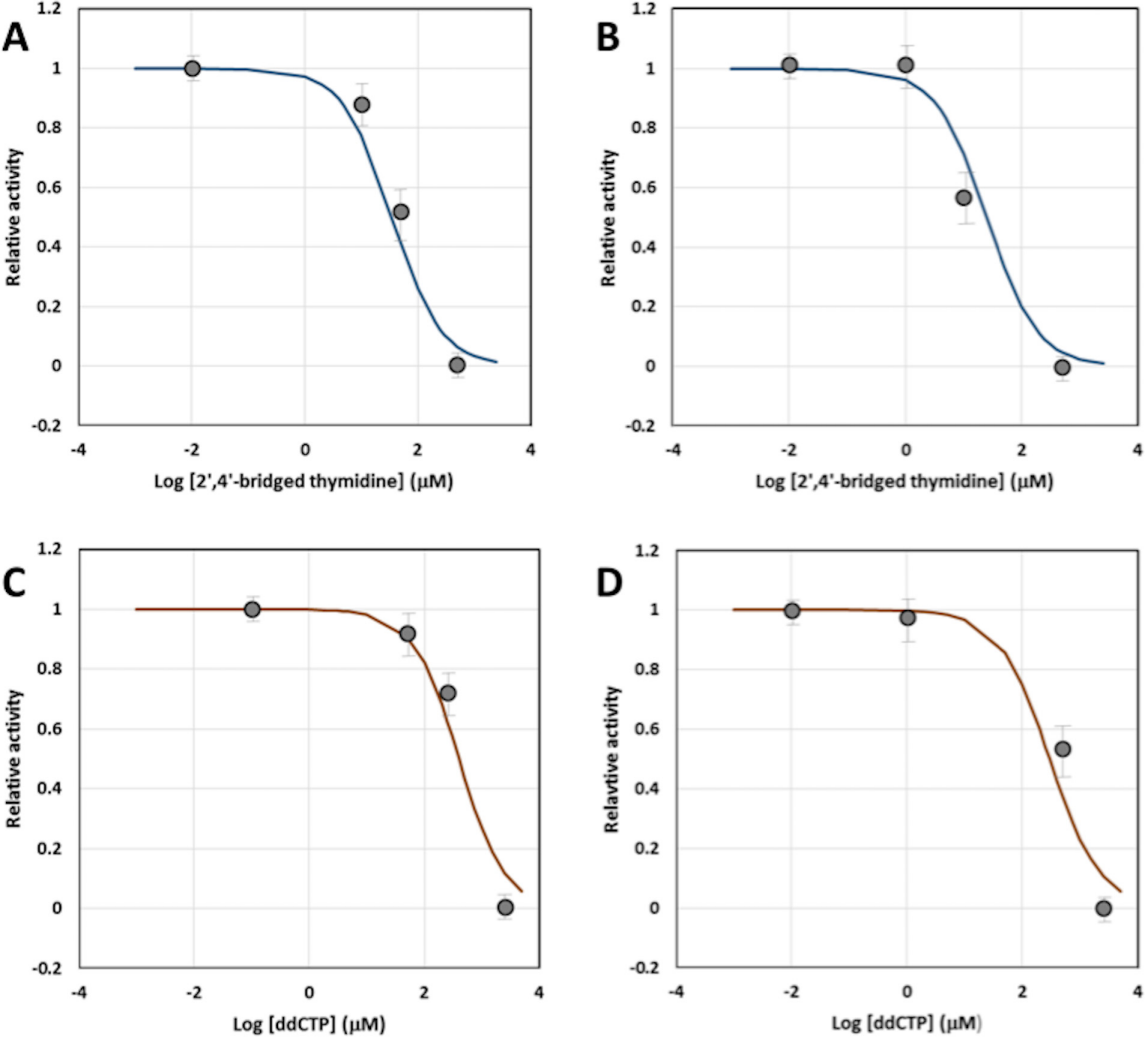
Determination of IC_50_ values. (A) IC_50_ value of *Taq* DNA polymerase by 2',4'-bridged thymidine using a primer extension assay with EvaGreen. The concentrations of 2',4'-bridged thymidine were used at 0.01, 10, 50, and 500 μM (B) IC_50_ value of *Taq* DNA polymerase by ddCTP using a primer extension assay with EvaGreen. The concentrations of 2',4'-bridged thymidine were used at 0.1, 50, 250, and 2500 μM (C) IC_50_ value of *Taq* DNA polymerase by 2',4'-bridged thymidine using the TaqMan method. The concentrations of 2',4'-bridged thymidine were used at 0.01, 1, 10, and 500 μM

To determine the inhibition mode of the 2',4'-bridged thymidine, we used various concentrations of the 2',4'-bridged thymidine and the substrate dNTP. Fig 3C shows a result of qPCR with various concentrations of dNTP ranging from 200 μM to 10 μM at a fixed 2',4'-bridged thymidine concentration of 5 μM, presenting a good separation in the difference of PCR products. The data was plotted as a double-reciprocal plot to clearly show the inhibition pattern of *Taq* DNA polymerase for the 2'-4'-bridged thymidine inhibitor (Fig 3D). As shown in Fig 3D, all the lines converge on the y-axis, indicating that the inhibition mode is a competitive mechanism with a *K*_i_ value of 9.7 ± 1.1 μM. In comparison to a known inhibitor of *Taq* DNA polymerase, iridoid catalpol, with an IC_50_ value of 47.8 μM [41], 2’,4’-bridged thymidine appears to be a promising inhibitor of *Taq* DNA polymerase. We also performed further experiments to support this TaqMan method to compare with a canonical primer extension assay. A sensitive fluorescent dye, EvaGreen, which binds to double stranded DNA, was used as a primer extension assay. First, the primer extension assay was employed to confirm the inhibitory effect by 2’,4’-bridged thymidine, and the results showed that the IC_50_ value was 35 ± 15 μM (Fig 4A and data in S1 Fig), which is in good agreement with the IC_50_ value (25 ± 10 μM) obtained by the TaqMan method (Fig 4B and data in S2 Fig). Additionally, we compared the two different methods with a known inhibitor of *Taq* DNA polymerase, ddCTP. The IC_50_ value obtained from the primer extension method was 400 ± 150 μM (Fig 4C and data in S3 Fig), and the IC_50_ value observed from the TaqMan method was 300 ± 100 μM (Fig 4D and data in S4 Fig). The fact that the inhibitory effect values quantified by the two different methods are consistent within the error range supports that the TaqMan method can be added to a repertoire of *Taq* DNA polymerase assays.

Molecular docking plays a pivotal role in structural biology and computational drug design. Because it can predict the binding interactions and affinities between ligands and their target molecules, molecular simulation is useful for a wide range of applications [42]. In order to investigate the binding between the nucleosides and *Taq* DNA polymerase, we used the most cited docking tool AutoDock Tools with AutoDock Vina, which enhances the average accuracy and computing process speed compared to standard Auto Dock [27]. Although the experimental data showed that among other nucleosides, 2',4'-bridged thymidine had significant inhibition by binding to the active site of the target protein, the simulation illustrated only slight differences in the binding affinity (ΔG) in the range between -5.9 and -7.4 kcal/mol (Table 1). Yet, a noticeable difference in hydrogen bonding interactions between the ligand and the protein could be observed, particularly near the Asp610 residue in the active site, suggesting the importance of Asp610 in the initial docking and succeeding inhibition.

**Table 1:** The structures, binding affinities and hydrogen bonds of ligands with the target molecule *Taq* DNA polymerase.

Ligand	Binding affinity (kcal/mol)	Amino acid involved in hydrogen bond
deoxyadenosine	-5.9	Glu615, Asp785, Glu832
thymidine	-5.9	Leu616, Lys663
5-methyldeoxycytidine	-5.9	Asp785, Glu832
deoxyguanosine	-6.8	Arg587, Glu615, Glu786, Glu832
2',4'-bridged adenosine	-6.8	Gln613, Ile614, Asp785, Glu786
2',4'-bridged thymidine	-6.7	Arg587, Asp610, Glu615, Glu786
2',4'-bridged 5-methylcytidine	-6.7	Gln613, Ile614, Glu786
2',4'-bridged guanosine	-7.4	Ile614, Glu615
2',4'-bridged (NC-NMe) adenosine	-7.0	Tyr611, Gln613, Ile614, Glu832
2',4'-bridged (NC-NMe) thymidine	-6.6	Ile614, Asp785
2',4'-bridged (NC-NMe) 5-methylcytidine	-6.8	Asp785, Glu786, Glu832
2',4'-bridged (NC-NMe) guanosine	-7.6	Arg587, Glu615, Glu786

**Fig 5:**
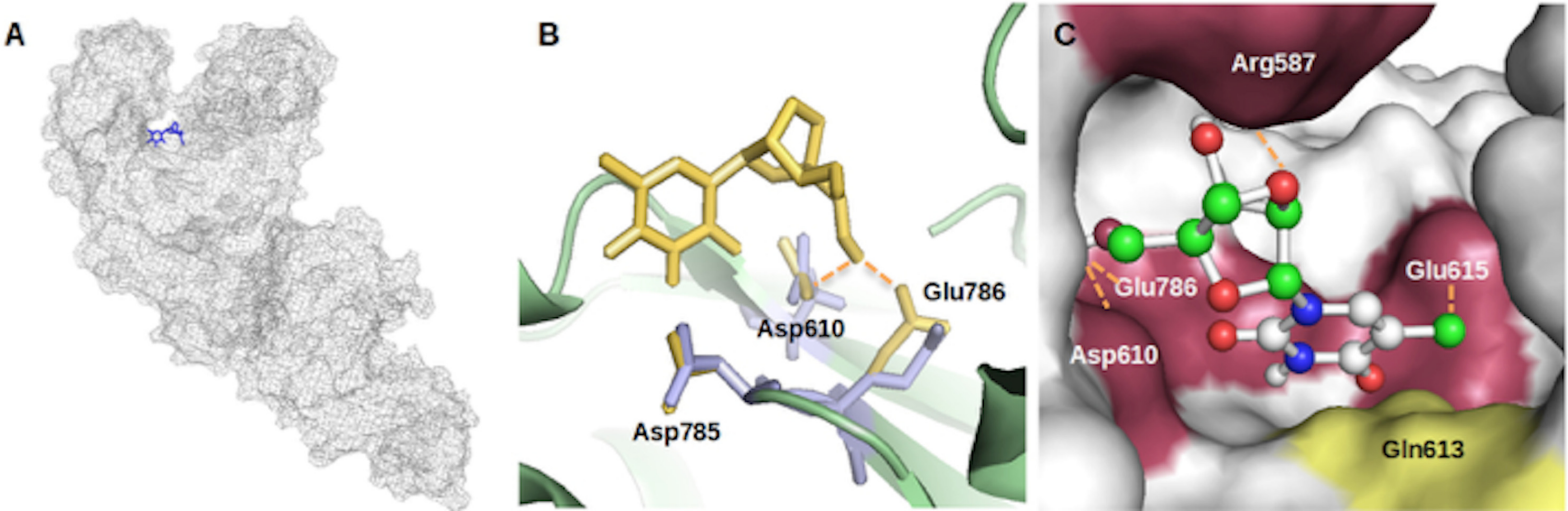
Molecular docking simulation of *Taq* DNA polymerase with 2',4'-bridged thymidine. (A) The whole structure of *Taq* DNA polymerase with 2',4'-bridged thymidine (blue color). (B) The overlapped structures of the catalytic sites (Asp610, Asp785, and Glu786) before and after molecular docking simulation. The residues before docking are colored in blue, and the residues after docking are colored in yellow. The dashed lines represent the hydrogen bonds. (C) The close view of the docking area with the hydrogen bonds. The residues involved in hydrogen bonds are colored in purple and the residue involved in van der Waals interaction is colored in yellow. The dashed lines represent hydrogen bonds.

As of binding location, it was clearly shown that 2’4’-bridged thymidine was well situated in the active pocket in the palm domain, providing additional evidence that the compound inhibits the enzyme in a competitive fashion (Fig 5A). Fig 5B and 5C show the docking results focused on the active site. A hydrogen bond with a distance of 2.6 Å formed between the oxygen of the carboxylate group in the Asp610 residue and the hydrogen of the hydroxyl group at the 5’ carbon of the ligand sugar ring. Hydrogen bonds also formed in other locations including Arg587 (where the bond formed between the hydrogen of the Arg587 guanidino group and the oxygen at the ligand 2’,4’-bridge), Glu615 (where the bond formed between the hydrogen of the Glu615 peptide bond and the oxygen at the 4 position of the base), and Glu786 (where the bond formed between the oxygen at the Glu786 carboxylate and the hydrogen of the hydroxyl group at the 5’ position of the sugar ring). It should be mentioned that X-ray crystallography revealed that the three residues, Asp610, Asp785 and Glu786, are the catalytic sites on the palm domain [28] and that the docking results revealed that 2’,4’-bridged thymidine was bound to two of the three catalytic sites (Asp610 and Glu786). Because the docking program allowed the assignment of a couple of residues in the flexible mode, three catalytic residues were set as flexible, reflected in Fig 5B.

**Fig 6:**
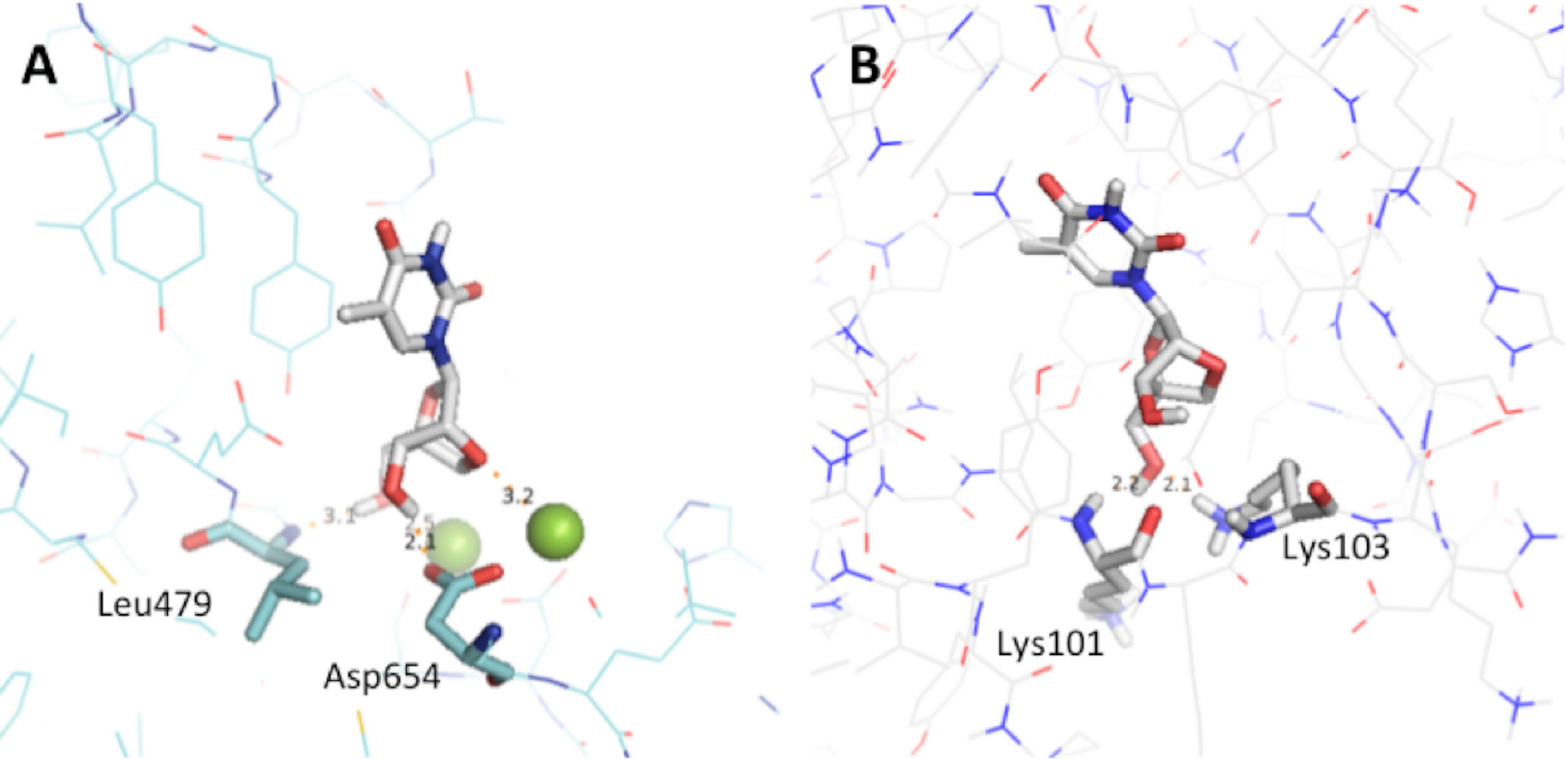
Molecular docking simulation of T7 DNA polymerase with 2',4'-bridged thymidine. (A) and HIV-1 RT with 2',4'-bridged thymidine (B). The hydrogen bonds between the protein and ligand are presented by dashed lines measured in angstrom unit. Oxygen atoms are colored in red; nitrogen in blue.

To explore the possibility of an inhibitory effect on other DNA polymerases, bacteriophage T7 DNA polymerase and HIV-RT were chosen as alternate receptors for molecular docking simulations with 2’,4’-bridged thymidine. In the case of T7 DNA polymerase, 2’,4’-bridged thymidine was clearly bound to the active site residues with a ΔG value of -6.4 kcal/mol, where four hydrogen bonds between 2’,4’-bridged thymidine and T7 DNA polymerase were formed (Fig 6A). 2’,4’-bridged thymidine formed hydrogen bonds with the two magnesium ions, which play an essential role in the catalytic mechanism of the enzyme. Additionally, the docking simulations showed hydrogen bonds with Asp654 and Leu479, residues located near the two metal ions in the active site. Thus, these observations reflect that 2’,4’-bridged thymidine may inhibit the bacteriophage T7 DNA polymerase. For HIV-1 RT, the docking results showed 2’,4’-bridged thymidine was situated in the active site with a ΔG value of -7.7 kcal/mol (Fig 6B). In this context, although there were no direct hydrogen bonds with one of catalytic sites of HIV-1 RT, all the catalytic sites were present in the vicinity of 2’,4’-bridged thymidine as shown in Fig 6B. Thus, based on our docking simulations for a viral DNA polymerase and a reverse transcriptase, it is not inconceivable that 2’,4’-bridged thymidine could be used as a potential drug for viral infections.

**Fig 7:**
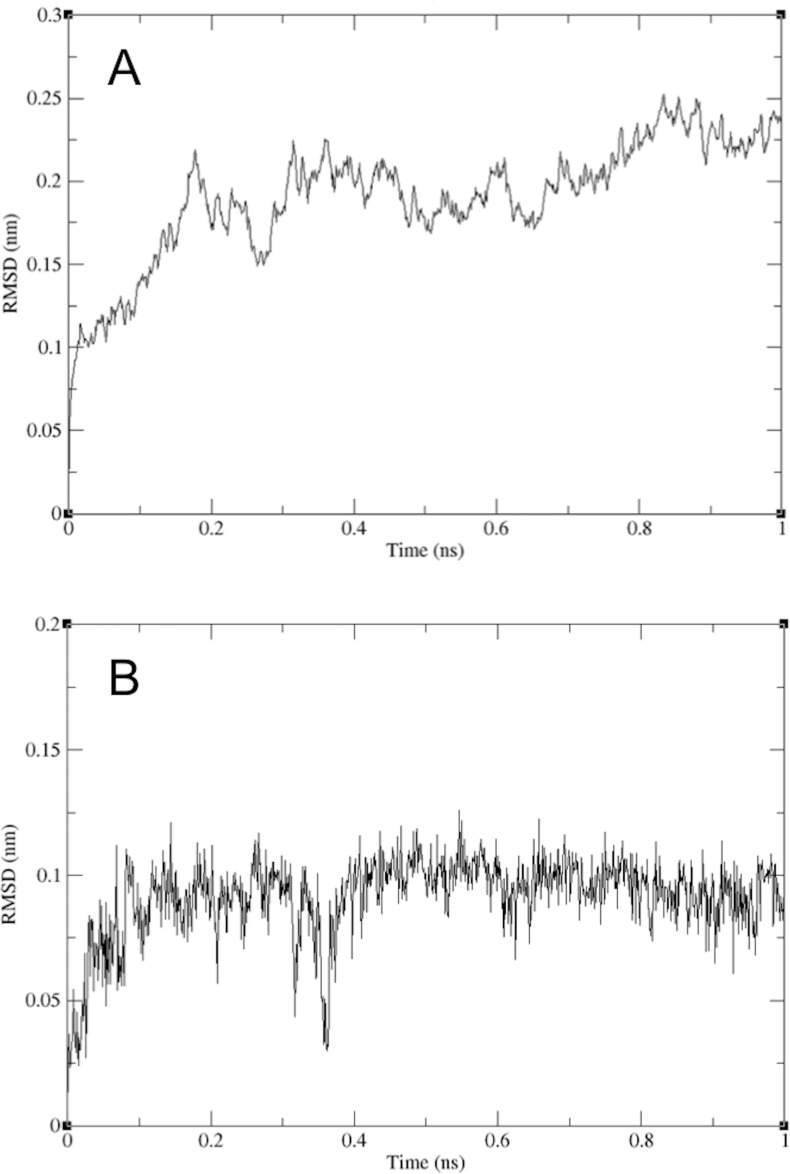
The RMSD derived from molecular dynamics simulations. (A) The RMSD of the complex peptide-backbone, *Taq* DNA polymerase and 2',4'-bridged thymidine. (B) The RMSD of the ligand 2',4'-bridged thymidine to *Taq* DNA polymerase.

In an effort to test conformational variations of the binding within a hydrated environment, MD simulations were carried out and the root-mean-square deviation (RMSD) of the atomic positions was analyzed. Fig 7A shows the RMSD for the peptide backbone of the enzyme as a function of the 1 ns simulation time. The obtained RMSD values varied around 2.3 Å and became stable after 0.8 ns, indicating that the molecular system was well behaved thereafter. The RMSD of the ligand was obtained under the same simulation conditions to get information on position fluctuations. As shown in Fig 7B, the RMSD of the ligand atoms rose to 1.0 Å after 0.2 ns and then leveled off. This indicates that after an initial increase in the magnitude of ligand atom fluctuation, the ligand reached an equilibrium state characterized by the RMSD profile.

**Fig 8:**
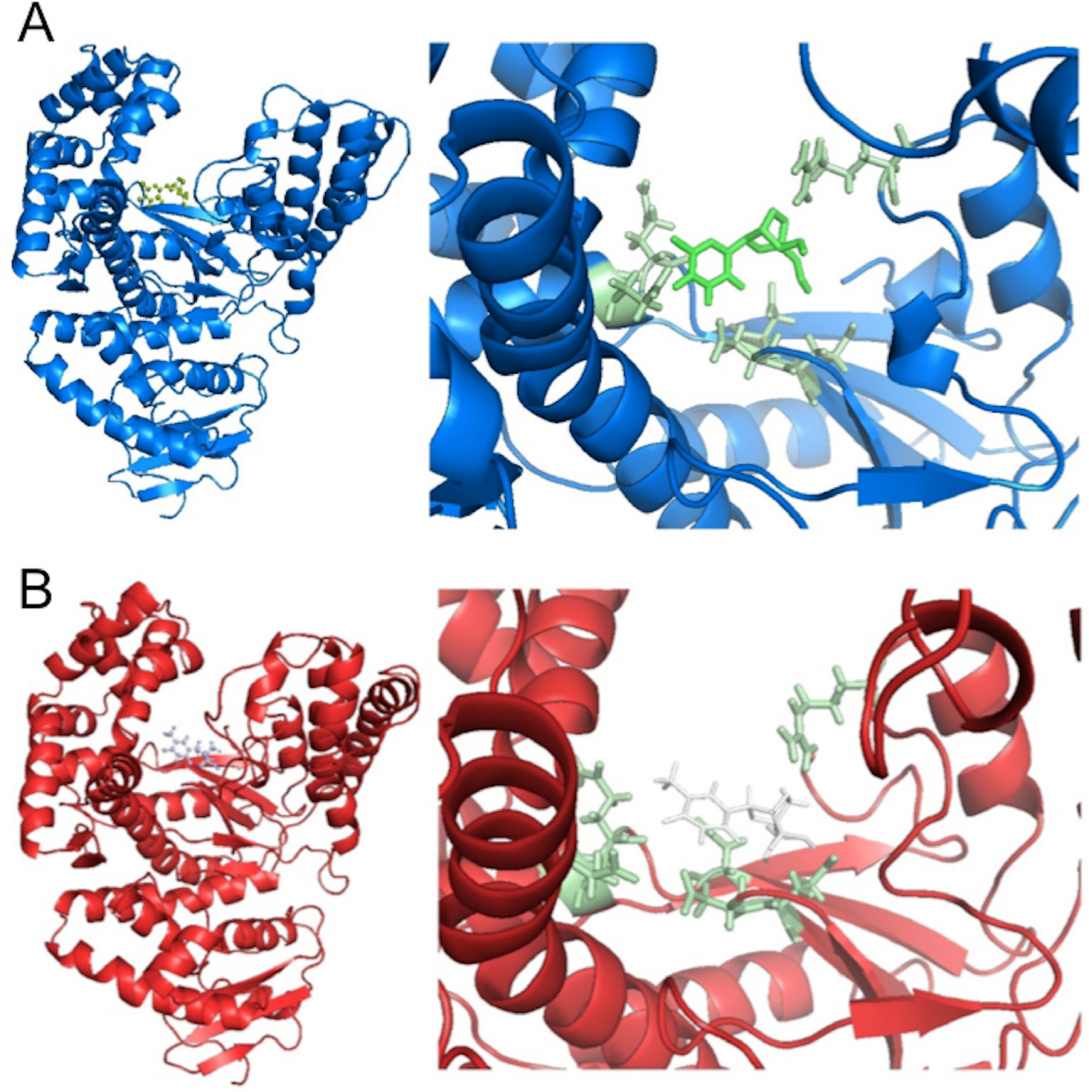
A snap shot of binding mode of 2',4'-bridged thymidine in *Taq* DNA polymerase. (A) before MD simulations (B) after MD simulations.

The analysis of the binding mode of the ligand obtained after MD simulation showed an approximate 40° rotation counter clockwise of the ligand with an approximate 45° rotation clockwise of the sugar moiety with respect to the initial state (Fig 8). Before the MD simulation, hydrogen bonds existed between 2’,4’-bridged thymidine and the residues Arg587, Asp610, Glu615 and Glu786. After the simulation, the hydrogen bonds with Asp610, Glu615 and Glu786 were broken and new hydrogen bonds (Leu609, Asp785 and Glu786) were formed. Arg587 appeared to have served as an axis of rotation for the ligand. It is important to note that the distance of each new hydrogen bond was significantly shorter than that of the hydrogen bonds before, suggesting that the new bonds are stronger. This observation lend insight to the reasons behind the effective inhibition of the enzyme by 2’,4’-bridged thymine. Moreover, the LNA monomer’s oxygen in the bridged moiety allows for interaction with guanidino group of Arg587 while the DNA equivalent’s lack of oxygen and the BNA equivalent’s bulky bridge structure prevented Arg587 and Glu786 interactions.

## Conclusions

Effort to find compounds that would inhibit *Taq* DNA polymerase focused on modified nucleosides containing a bridge on the sugar moiety. As a result, the compound 2’,4’-bridged thymidine was found to effectively inhibit the polymerase in a competitive fashion. The observation could possibly spark a great deal of interest in such bridged nucleosides as lead compounds. In addition, a novel assay method was used to determine kinetic parameters using qPCR. Particularly, for the initial inhibition tests, the use of the SYBR Green dye to probe the inhibition possibility and the subsequent use of TaqMan to gain the information about kinetic parameters combine to facilitate a facile method of study for any *Taq* DNA polymerase inhibition experimentation. Docking analysis has provided the insight into the initial docking by the ligand 2’,4’-bridged thymidine to the active site of the enzyme. The fact that the Asp610 residue was a unique binding site for 2’,4’-bridged thymidine compared to other nucleosides docking sites provides an idea that the residue appears to be a critical binding site. The MD simulations supported the idea that the ligand was still stabilized inside the active pocket. The hydrogen bond between the residue Arg587 of the enzyme and the ligand appeared to be the most important anchoring and pivotal interaction, allowing the ligand to twist into its most stable state. From these observations, we can conclude that the *in silico* analyses not only soundly supported the experimental data but also provided indispensible knowledge of how the inhibitor binds to the enzyme.

## Supporting Information

S1 Fig The profiles of the primer extension assays by EvaGreen with various concentrations of 2’4’-bridged thymidine. A primer extension assay was carried out at the 2’4’-bridged thymidine concentrations of 0.01 μM (A), 10 μM (B), 50 μM (C), and 500 μM (D).
(TIFF)

S2 Fig A console outcome of qPCR using 2',4'-bridged thymidine at various concentrations. The line colored in yellow represents 500 μM 2',4'-bridged thymidine; the line colored in pink denotes 10 μM 2',4'-bridged thymidine; the lines colored in purple are 1 μM (bottom) and 0.01 μM (top) 2',4'-bridged thymidine compounds.
(TIFF)

S3 Fig The profiles of primer extension assays by EvaGreen with various concentrations of ddCTP. A primer extension assay was carried out with ddCTP concentrations of 0.01 μM (A), 250 μM (B), 500 μM (C), and 2.5 mM (D).
(TIFF)

S4 Fig A console outcome of qPCR using ddCTP at various concentrations. The line colored in purple represents 2500 μM ddCTP; the line colored in red denotes 500 μM ddCTP; the lines colored in green are 1 μM (bottom) and 0.01 μM (top) ddCTP.
(TIFF)
