# Supplementary figures and images for: Inhibitory Effect of Bridged Nucleosides on Thermus aquaticus DNA Polymerase and Insight into the Binding Interactions
Source: PLoS One. 2016 Jan 28;11(1):e0147234. doi: 10.1371/journal.pone.0147234 (PMC4731470; doi:10.1371/journal.pone.0147234)

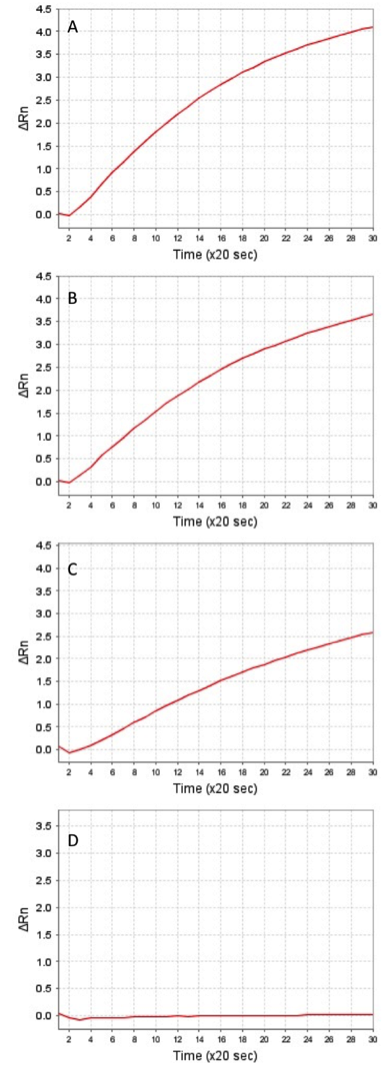

Supplement: S1 Fig — A primer extension assay was carried out at the 2’4’-bridged thymidine concentrations of 0.01 μM (A), 10 μM (B), 50 μM (C), and 500 μM (D). (TIFF) [file pone.0147234.s001.tiff]

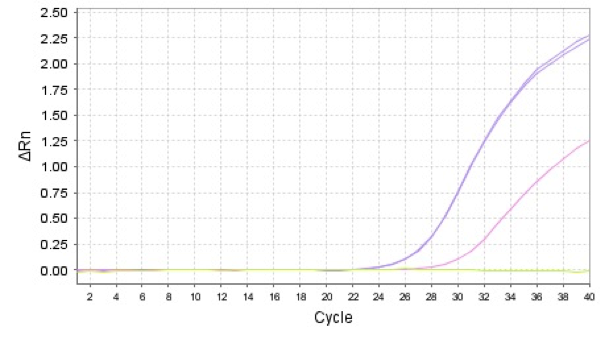

Supplement: S2 Fig — The line colored in yellow represents 500 μM 2',4'-bridged thymidine; the line colored in pink denotes 10 μM 2',4'-bridged thymidine; the lines colored in purple are 1 μM (bottom) and 0.01 μM (top) 2',4'-bridged thymidine compounds. (TIFF) [file pone.0147234.s002.tiff]

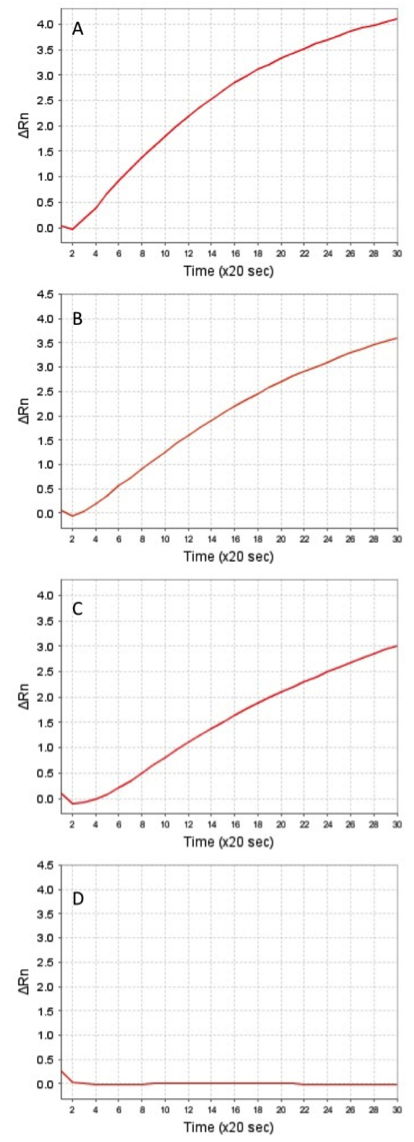

Supplement: S3 Fig — A primer extension assay was carried out with ddCTP concentrations of 0.01 μM (A), 250 μM (B), 500 μM (C), and 2.5 mM (D). (TIFF) [file pone.0147234.s003.tiff]

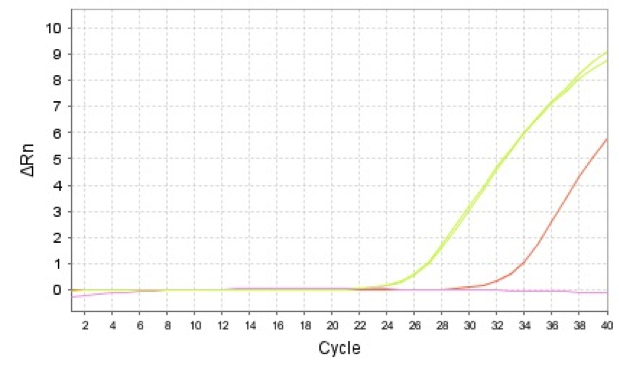

Supplement: S4 Fig — The line colored in purple represents 2500 μM ddCTP; the line colored in red denotes 500 μM ddCTP; the lines colored in green are 1 μM (bottom) and 0.01 μM (top) ddCTP. (TIFF) [file pone.0147234.s004.tiff]
